# An audit on sensitive documentation of suicide attempts and behaviour in Mount Carmel Hospital discharge summaries

**DOI:** 10.1192/j.eurpsy.2025.908

**Published:** 2025-08-26

**Authors:** N. Borg, R. Gambin, G. Gatt, F. Cassar

**Affiliations:** 1 Mental Health Services, Attard; 2 Mater Dei Hospital, Msida, Malta

## Abstract

**Introduction:**

Suicide attempts should always be recorded in a patient’s discharge summary as this aids risk assessment and management. Discharge letters provide valuable information to outpatient and emergency services and guide risk assessment. However, they are available to be read by patients and their loved ones. Insensitive wording or graphic detail can contribute to feelings of shame and guilt and perpetuate stigma towards our patients.

**Objectives:**

This audit aimed to assess quality of documentation of suicide attempts and behaviour on Mount Carmel Hospital (MCH) discharge summaries with respect to the International Association of Suicide Prevention (IASP)’s Language guidelines. It also aimed to assess the effectiveness of a short presentation intervention on suicide given to foundation (FY) doctors.

**Methods:**

For the first cycle, discharge letters of patients admitted to MCH between January and July 2023 were screened according to inclusion criteria. Included discharges were assessed for adherence to IASP’s language guidelines. The authors then gave a short presentation to FY doctors during their rotation, highlighting the importance of using sensitive language on suicide in discharge summaries. The second cycle was carried out between January and July 2024 and results were compared to the first cycle.

**Results:**

In the first cycle, a total of 1393 patients were admitted between January and July 2023. 26% of discharge letters did not use appropriate suicide-related terminology. Of these, the most common issues were with excessive description of the attempt (53%) and use of the phrase ‘commit suicide’ (28%). In the second cycle, a total of 1335 patients were admitted between January and July 2024. 20% of discharges did not use appropriate suicide-related terminology (n = 34). The intervention significantly reduced the use of the phrase ‘commit suicide’ (OR 0.279; CI 0.0921 to 0.8452, p = 0.0240).

**Conclusions:**

In the first cycle, one out of every four patients admitted on a background of suicidal behaviour received a discharge summary that it potentially insensitive or stigmatising in nature. This decreased to one in five after the short intervention. These results are promising especially given that the intervention is neither time consuming nor costly. Simply making foundation year doctors aware and guiding them on how to write about suicide can not only improve the quality of patient care but also reduce iatrogenic harm. Additionally, foundation doctors should be supported and supervised by more senior firm members to ensure that discharge letters are not only of good quality but also written sensitively and sensibly.

**Disclosure of Interest:**

None Declared

